# Explainable AI to unveil cellular autophagy dynamics

**DOI:** 10.1371/journal.pone.0331045

**Published:** 2025-09-11

**Authors:** Oriana Presacan, María Hernández Mesa, Alexandru C. Aldea, Siri Andresen, Amani Al Outa, Julie Aarmo Johannessen, Bogdan Ionescu, Helene Knævelsrud, Michael A. Riegler

**Affiliations:** 1 AI Multimedia Lab, Campus Research Institute, National University of Science and Technology Politehnica Bucharest, Bucharest, Romania; 2 Department of Molecular Medicine, Institute of Basic Medical Sciences, University of Oslo, Oslo, Norway; 3 Centre for Cancer Cell Reprogramming, Institute of Clinical Medicine, Faculty of Medicine, University of Oslo, Oslo, Norway; 4 Department of Molecular Cell Biology, Institute for Cancer Research, Oslo University Hospital, Oslo, Norway; 5 Faculty of Biotechnologies, University of Agronomic Sciences and Veterinary Medicine, Bucharest, Romania; 6 Simula Research Laboratory, Oslo, Norway; Nathan S Kline Institute, UNITED STATES OF AMERICA

## Abstract

Autophagy is a fundamental intracellular renovation process vital for maintaining cellular homeostasis through the degradation and recycling of damaged components. It is implicated in numerous pathological conditions, including cancer and neurodegenerative diseases. However, its dynamic nature and complexity pose challenges for manual analysis. In this study, we present a computational pipeline that leverages advanced deep learning models to automate the analysis of autophagic processes in 6,240 fluorescence microscopy images from the CELLULAR dataset. Our framework integrates object detection, cell segmentation, classification by autophagic state, cellular tracking, and explainability methods for interpretability. We achieved optimal results using YOLOv8 for object detection with a mAP50 of 0.80, U-Net++ for segmentation with an IoU of 0.82, and a vision transformer for classification with an accuracy of 0.86. To track cells, we developed a custom algorithm capable of handling complex scenarios such as cell division and morphological changes, all without requiring annotated tracking data. To enhance transparency, we employed explainability techniques based on class activation mappings to analyze model decision-making processes and validate classification outcomes, complemented by t-SNE visualizations for deeper insights into the data. Collaboration with biology experts validated our findings, highlighting the pipeline’s potential to advance autophagy research. This study demonstrates the potential of deep learning and explainable AI to streamline biomedical research, reduce manual effort, and uncover key autophagy dynamics.

## Introduction

Autophagy is a continuous intracellular process of degradation and recycling conserved across all eukaryotic organisms, including humans. It plays an essential role in maintaining cellular homeostasis by turning over damaged or unnecessary cellular components. Under conditions of stress, such as nutrient deprivation and pathogenic infections, this function becomes vital [1]. The process can be classified into three distinct types: microautophagy, macroautophagy, and chaperone-mediated autophagy, each characterized by unique mechanisms for delivering cellular cargo to the lysosome for degradation [2]. In this study, we focus specifically on macroautophagy, herein referred to simply as autophagy, due to its predominant role in intracellular degradation.

Autophagy impacts various physiological processes, influencing both health and disease. Dysregulation of autophagy has been linked to numerous pathological conditions, including cancer [3], neurodegenerative disorders [4], and infections [5]. For instance, defective autophagy in neurodegenerative disorders leads to the accumulation of toxic protein aggregates, worsening disease progression [4]. In cancer, autophagy plays a multifaceted role, capable of both promoting and inhibiting tumor growth depending on the tumor’s stage and influencing immune system responses [6]. This dual nature makes it imperative to understand how to correctly target autophagy for cancer treatment. A study on autophagy’s role in cancer [7] highlights the need to refine strategies for leveraging this complex process, emphasizing the importance of understanding its molecular mechanisms to develop effective therapies.

**Fig 1:**
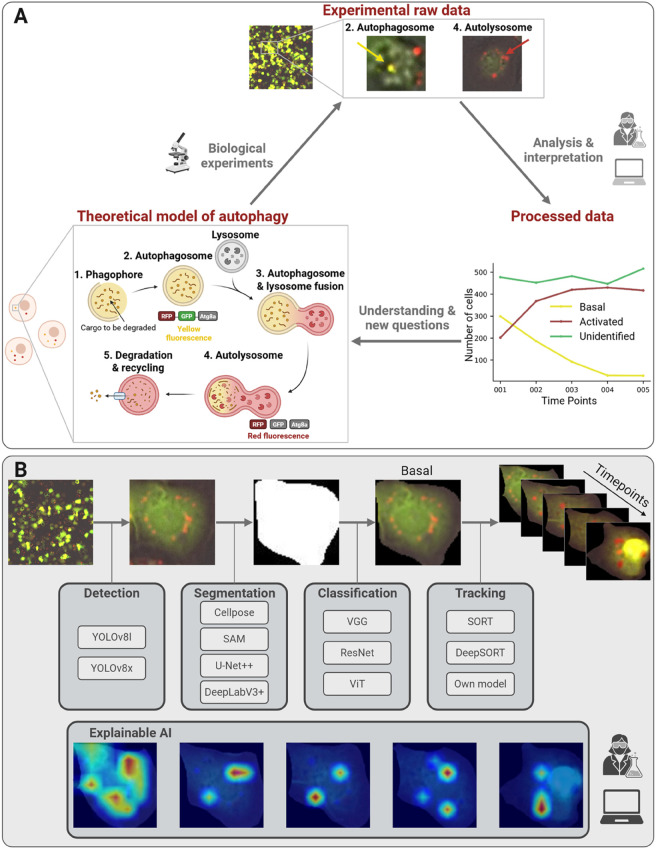
Overview of our deep learning pipeline for analyzing cellular autophagy. Panel A: Biological experiments generate raw imaging data of Drosophila melanogaster S2 cells under different conditions. A theoretical model of autophagy illustrates key cellular stages, guiding the analysis. Processed data, derived from computational methods, provides insights into autophagy dynamics, enabling further interpretation. Panel B: Our computational pipeline follows five steps: (1) Detection to identify cells, (2) Segmentation to isolate structures, (3) Classification to categorize autophagy states, (4) Tracking to monitor cell dynamics, and (5) Explainable AI to enhance interpretability and biological relevance.

The autophagic process involves the identification, degradation, and recycling of damaged, superfluous, or aggregated cellular components. It begins with the development of the phagophore, a membrane structure that envelops the material to be broken down (see step 1 in the inset of Fig 1). This phagophore elongates and closes to form a double-membraned vesicle called an autophagosome. The autophagosome then fuses with a lysosome, an organelle rich in digestive enzymes, forming an autolysosome. Within the autolysosome, the enclosed material is broken down, and the degraded components are recycled to the cell cytoplasm for reuse [1].

To study these dynamic and transient structures, researchers rely on fluorescence microscopy, which enables the real-time visualization and tracking of autophagosomes and autolysosomes during the autophagic process [8]. A tandem tag of red and green fluorescent proteins is often fused to specific markers involved in autophagy, such as proteins from the Atg8 family, thus facilitating differentiation between autophagosomes and autolysosomes [9]. Consequently, studying autophagy based on microscopy images involves identifying cells, determining the presence of autophagosomes and autolysosomes, and evaluating the autophagic state of the cells. However, given the complexity of experiments that span hours under diverse physiological conditions and involve large volumes of images, manual analysis becomes tedious and inefficient.

Despite the importance of autophagy in health and disease, its analysis remains challenging due to the complex and dynamic nature of the process. Traditional manual methods, such as manual quantification and thresholding, are labor-intensive, prone to variability, and unsuitable for large-scale datasets under diverse experimental conditions. Moreover, there is a lack of automated tools specifically designed for analyzing high-resolution autophagy images, limiting the ability to accurately identify distinct autophagic states and comprehensively track cellular dynamics.

Advancements in artificial intelligence (AI) offer promising solutions to address these challenges. While deep learning has been widely applied across various biological domains, its use in autophagy research remains limited [10]. This is likely due to the limited availability of extensive, annotated datasets required for training deep learning models. As a result, deep learning integration in autophagy research is still in its early stages, revealing a gap between AI’s potential and its current application in the field.

To bridge this gap, our study leverages deep learning models to analyze cellular autophagy using image-based data. As a case study, we utilize the CELLULAR dataset [11], which consists of images of *Drosophila melanogaster* S2 cells expressing mRFP-EGFP-Atg8a under distinct nutritional conditions. Our approach begins with statistical analyses to extract foundational insights from the dataset. We then apply deep learning techniques for cell segmentation and classification into three categories: basal autophagy, activated autophagy, or an unidentified category. Additionally, we track cellular dynamics across different conditions to capture variations in autophagic activity. To enhance interpretability, we leverage explainable AI (XAI) methods to identify key patterns and factors influencing model predictions. This interpretability step ensures that our findings align with biological expectations, a process further validated through collaboration with domain experts. The workflow of our proposed system is depicted in Fig 1.

## Materials and methods

### Data

#### CELLULAR dataset.

**Fig 2:**
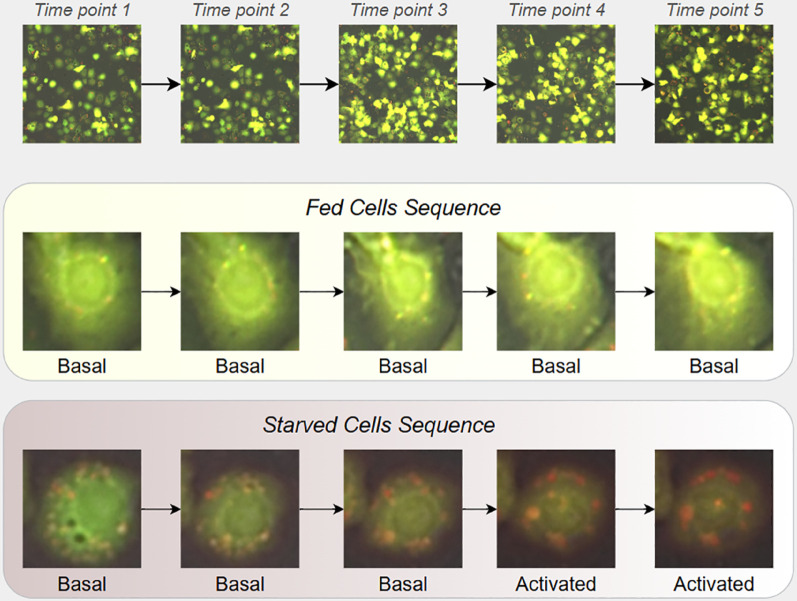
Time-lapse imaging from the CELLULAR dataset. The top panel shows progressive changes in a cell population over five time points. The middle panel (Fed Cells Sequence) depicts an individual fed cell remaining in a basal autophagy state throughout the time course. The bottom panel (Starved Cells Sequence) shows a starved cell transitioning from a basal to an activated autophagy state.

The CELLULAR dataset [11] is a publicly available collection of fluorescence microscopy images of *Drosophila melanogaster* S2 cells, cultured under nutrient sufficiency (“fed”) and nutrient deprivation (“starved”). To monitor autophagy, cells were genetically engineered, as described in [12], to express the mRFP-EGFP-Atg8a reporter [9], consisting of monomeric red fluorescent protein (mRFP), enhanced green fluorescent protein (EGFP), and Atg8a protein. Under neutral pH conditions, both fluorescent proteins emit light and produce red and green fluorescence, respectively. However, in acidic environments, EGFP is quenched, yielding only red fluorescence. This differentiation allows the identification of autophagosomes (exhibit both green and red fluorescence, appearing yellow) and autolysosomes (red fluorescence only). Images were captured hourly over a four-hour period, producing sequences of five images per sample to document the progression of autophagy. Fig 2 shows an example sequence, highlighting the transition from basal to activated autophagy states in starved cells, contrasted with stable basal autophagy states in fed cells.

The dataset consists of 18,720 TIF files, with each file corresponding to a channel (green, red, transmitted light) across 6,240 samples. Among these, 53 images have been annotated by human experts. For the annotated images, merged color images at a resolution of 2048x2048 pixels are provided, along with segmentation masks, one mask per cell, bounding boxes, and class labels. Cells are classified according to their autophagy status into three categories: basal autophagy, activated autophagy, and unidentified. For this study, only the 53 annotated color images and their corresponding annotations were used.

The dataset, consisting of 53 images, was divided into training, validation, and test sets in a 35:8:10 split (train:validation:test). The test set includes two complete sequences: one of fed cells and one of starved cells. A file listing the exact filenames used for training, validation, and testing is available in our GitHub repository (data_division.txt).

We analyzed annotated images to understand their content and temporal evolution. Each image contains between 140 and 386 cells, with 44.9% unidentified, 32.8% showing basal autophagy, and 22.8% with activated autophagy. We analyzed transitions between cell classes over the five time points under fed (4 sequences) and starved (3 sequences) conditions. Only complete image sets with annotations for all five time points were included. Under starvation, basal autophagy cells decreased while activated autophagy cells increased. In fed conditions, most cells consistently showed basal autophagy (see S1 Table in Supplementary Material).

Using segmentation masks, we analyzed cell morphology, focusing on basal and activated cells. Cell area and circularity were calculated from pixel counts, revealing that basal autophagy cells are 12.65% larger and 5.97% less circular than activated autophagy cells. A t-test confirmed significant differences in both metrics (see S2 Table in Supplementary Material).

#### RNA interference (RNAi) dataset.

For the RNAi dataset, 2000 S2 mRFP-EGFP-Atg8a cells in ESF921 medium were cultured with 4000 ng dsRNA against Atg1, mTor or Luciferase and incubated at 25°C for five days. Cells were imaged in a 384-well glass-bottom plate (Cellvis, P384-1.5H-N) pre-coated with 0.5 mg/mL concanavalin A (Sigma, L7647). Live-cell time-lapse imaging was conducted at room temperature using an ImageXpress Micro Confocal High-Content Imaging System (Molecular Devices). Imaging was performed in wide-field acquisition mode, capturing three different visiting points per well with a 40× Plan Fluor ELWD air objective. For each field, images were collected in three channels: green fluorescence (GFP), red fluorescence (RFP), and transmitted light (TL). Live-Cell Imaging Solution (Molecular Probes, A14291DJ) supplemented with 2 mg/mL glucose (Formedium, GLU03) was used to induce starvation and the cells were followed over time.

### Traditional experimental analysis

#### Western blot.

S2-mRFP-EGFP-Atg8a were either kept fed in Schneider’s Drosophila Medium (GIBCO 21,720,001) supplemented with 10% heat-inactivated FBS (GIBCO, F7524) and 1% penicillin-streptomycin (GIBCO 15,140–122) or subjected starvation by using 1X phosphate-buffered saline (PBS; Gibco 14,190–094) supplemented with 2 mg/mL D(+)glucose (Formedium, GLU03). At the end of the treatment period, cell lysates were prepared using ice cold RIPA Buffer (50 mM Tris-HCl, pH 7.4, 150 mM NaCl, 0.25% deoxycholic acid, 1% NP-40, and 1 mm EDTA; Millipore, 20–188) supplemented with phosphatase (Roche 04,906,837,001) and protease (Roche 05,056,489,001) inhibitors. The cell lysates were centrifuged for 15 min at 14,000×g at 4°C before the supernatant was transferred to a new tube while the pellet was discarded. Supernatant was used for protein quantification using the bicinchoninic acid (BCA) Protein Assay Kit (Thermo Fisher 23,227). Protein extract was mixed with 4X sample buffer (Thermo Fisher, NP0008) and 10X 1 M DTT (Sigma-Aldrich, D0632) to a final concentration of 1X and boiled for 5 min at 95°C. The samples were separated by SDS-PAGE on 4–20% gradient gels (Bio-Rad, 567–1094, 567–1095). Proteins were then transferred onto LF PVDF membrane (Bio-Rad, 161–0374) using the semi-dry Trans-Blot^®^ Turbo^™^ Transfer System (Bio-Rad 1,704,150). The membrane was air dried for 15 min before incubation with the primary antibodies rabbit anti-*β*-actin/Act5C 1:1000 (Abcam, ab8227) and goat anti-mCherry 1:500 (Acris, AB0040–200) and rotated overnight at 4°C. Next day, the membrane was washed three times for 10 min in Tris-buffered saline (TBS; Bio-Rad 10,026,938) with 0.1% Tween 20 (Sigma-Aldrich, P1379), and then incubated with the horseradish peroxidase (HRP)-conjugated secondary antibodies anti-rabbit 1:5000 (Jackson, 111-035-144) and anti-goat 1:5000 (Jackson, 705-035-147) in TBS-Tween 20 with 5% skim milk powder (Millipore 70,166) for 1 h at room temperature. The antibodies were detected by chemiluminescence, using SuperSignal West DURA Extended Duration Substrate (Thermo Fisher 11,593,440) and captured using ChemiDoc MP system (Bio-Rad).

#### Flow cytometry.

EGFP and mRFP expression levels were assessed in fed and starved mRFP-EGFP-Atg8a S2 cells by flow cytometry. Prior to analysis, the cells were centrifuged twice at 500 g for 5 minutes before being resuspended in 1 mL IPL-41 media (Sigma, I7760) for fed condition or 1 mL Live-cell imaging solution (Molecular probes, A14291DJ) supplemented with 2 mg/mL glucose (Formedium, GLU03) for starved condition. Hoechst (Hoechst 33258, Thermo Fisher, H3569) was added at a final concentration of 0.008 μg/mL to enable removal of dead cells during analysis. Cell suspensions were subsequently passed through 35 μm filter cap tubes (Falcon, 352235) before analysis on an LSR II flow cytometer (BD). Laser 488 nm with bandpass filter 525/30 nm and longpass Dichroic filter 505 nm was used to detect EGFP, and laser 561 nm with bandpass filter 582/15 nm and longpass Dichroic filter 570 nm was used to detect mRFP. Approximately 20.000 events (cells) were used for the analysis using the software FlowJo v10 (BD). S2 cells with no reporter expression were used as a negative control.

### Traditional image analysis

To analyze the dataset with traditional image analysis, CellProfiler (version 4.2.6) [13] was used to segment RFP spots and extract cell features using the ground truth as input for cell masks. Cells where the CellProfiler pipeline failed to segment RFP spots were excluded from further analysis. Feature values were normalized within experiment replicates and subsequently log10 transformed to achieve a normal distribution. A threshold for activated autophagy was established based on the 95th percentile of RFP spot area from fed cells at timepoint 1. Cells with RFP spot areas exceeding this threshold were classified as having activated autophagy. This classification was then compared against the ground truth annotations in the CELLULAR dataset. All analyses for the conventional image analysis were conducted in R (version 4.3.1)/RStudio (version 2025.05.1) and the associated code is available on GitHub.

### Deep learning analysis

#### Hardware specifications.

We trained the deep learning models using an NVIDIA GeForce RTX 4080 Super GPU with 16GB of VRAM and an i9-13900K processor and 128GB of RAM. The code was written in Python 3.12 and the models were implemented using the PyTorch 2.5 framework with CUDA support. For each of the models below, a requirements file specifying the required package versions has been provided in the GitHub repository.

#### Object detection.

Object detection is the process of identifying and categorizing objects within images or video. In this context, it involves detecting the presence of cells and determining their positions, depicted by rectangular bounding boxes. For the detection task, we used the well-established You Only Look Once (YOLO) model [14]. Given the successful applications of YOLO models in related works [15], the state-of-the-art YOLOv8 model was selected for this study.

Two pre-trained YOLOv8 models of different sizes were utilized: YOLOv8x (extra-large) with 67 million parameters and YOLOv8l (large) with 43 million parameters. Both models were fine-tuned with a batch size of 4, preserving the original 2048×2048 resolution and using YOLO’s default augmentations, including flipping, scaling, rotation, and others. The training was carried out until the validation loss stagnated for 50 consecutive epochs, after which the model checkpoint with the best performance was saved.

#### Segmentation.

Cell segmentation is the process of isolating cells from the background to enable further morphological analysis. This involves classifying each pixel in the image into cell or no-cell classes based on its characteristics. Initially, instance segmentation of entire cell images was prioritized, treating all cells uniformly without autophagic class differentiation.

We selected two models: the Segment Anything Model (SAM) [16], recognized for its remarkable performance and generalizability in instance segmentation tasks [17]; and Cellpose 2.0 [18], a widely used framework specifically designed for cell segmentation. Both were fine-tuned on the CELLULAR dataset. For SAM, we followed a community guide [19] targeting only the decoder and using the pre-trained vit_h model. Images were resized to 1024×1024 pixels. The training was conducted over 100 epochs with a batch size of 4, using the Adam optimizer (learning rate: 0.001, weight decay: 0.0005), and combined focal and dice loss [16]. For Cellpose 2.0, we fine-tuned the cyto2torch3 model using existing [11]. This also spanned 100 epochs with a batch size of 4, using the SGD optimizer (learning rate: 0.2, weight decay: 0.00001), and combined mean squared error (MSE) and binary cross-entropy (BCE) loss.

Due to frequent misidentification by these models, we transitioned to integrating segmentation with object detection for better control (see Section Object Detection). Object detection models with adjustable confidence thresholds allow for higher accuracy by isolating and subsequently segmenting detected cells. SAM was selected again for this task due to its superior performance compared to Cellpose 2.0. In addition, the DeepLabV3+ and U-Net++ models were included for comparison. Both utilized a pre-trained ResNet50 encoder and were trained with normalized images, a batch size of 32, dice loss, the Adam optimizer (learning rate: 0.0001), a learning rate scheduler (patience: 10 epochs), and early stopping (patience: 30 epochs).

We used a custom test script, available in our GitHub repository, to ensure consistent model evaluation on the test dataset. For SAM, we assessed both pre-trained and fine-tuned models, while DeepLabV3+ and U-Net++ evaluations were based on versions trained from scratch on the CELLULAR dataset.

#### Classification.

This study classifies cells according to their autophagy status: basal autophagy, activated autophagy, or unidentified. We employed well-established architectures such as VGG-16, ResNet50, and Vision Transformer (ViT) due to their proven performance in image classification [20–22].

Our strategy involved both training these models from scratch and fine-tuning models pre-trained on large datasets like ImageNet [23]. We also compared the use of cropped images containing a single cell per image with segmented images where cells are isolated against a black background to evaluate the impact on model performance. VGG-16, ResNet50, and ViT models were obtained from the *torchvision.models* subpackage, adapting their final linear layer for 3-class classification. The images were normalized using the dataset’s mean and standard deviation and resized to 224x224 pixels. Training employed a batch size of 32, cross-entropy loss, the Adam optimizer (learning rate: 0.0001), a learning rate scheduler reducing the learning rate if validation loss did not improve over 10 epochs, and early stopping if no improvement was seen over 30 epochs, with checkpoints saved for the lowest validation loss.

#### Tracking.

Tracking is essential for monitoring individual cells’ progression across the five frames in our time-lapse dataset. This allows the analysis of cellular responses to nutritional factors like starvation or nutrient presence. However, the CELLULAR dataset lacks tracking annotations, posing a significant challenge.

We explored several object tracking algorithms, focusing on two widely used multiple object tracking methods: SORT [24] and DeepSORT [25], the latter being an extension of SORT. Building on these, we developed a custom algorithm specifically tailored to our dataset. Our method utilized the Hungarian algorithm for data association while omitting Kalman filters due to the limited number of frames. To extract feature vectors, it leveraged a pre-trained ResNet50 encoder. The algorithm distinguished between active (currently tracked) and inactive cells (previously tracked but currently undetected), preserving historical data for potential re-detection and addressing inconsistencies in human annotations. However, due to the absence of ground truth annotations for tracking, we are unable to quantitatively assess the performance of the models.

#### Explainability.

Deep learning models perform complex calculations, making them difficult to interpret, which raises trust concerns, especially in sensitive fields like healthcare [26]. Explainable AI (XAI) addresses this by developing techniques to enhance model interpretability. One widely used XAI method is Class Activation Mapping (CAM) [27], designed for convolutional neural networks (CNNs) to highlight key input features influencing predictions via heatmaps. However, CAM requires a specific architecture, limiting its applicability. To overcome this, we selected three CAM extensions—GradCAM [28], EigenCAM [29], and AblationCAM [30]—each offering a unique approach to importance calculation. EigenCAM applies principal component analysis (PCA) to feature maps, capturing dominant patterns without relying on a specific class score. GradCAM uses output gradients to identify regions contributing to a prediction, while AblationCAM determines importance by systematically removing feature map components and measuring the impact.

Although initially designed for CNNs, these methods can be adapted for transformers. We modified the [31] repository to suit our ViT model, which achieved the highest classification accuracy. In transformers, selecting the target layer for Grad-CAM is crucial since decisions rely on the class token in the final attention block. As the last layer lacks gradients, we used the preceding one layer_11.ln_1 layer for visualization. Model outputs were reshaped from [batch, 50, 768] to exclude the class token, focusing on the 7x7 image patches with 768 channels.

#### tSNE.

t-Distributed Stochastic Neighbor Embedding (t-SNE) is a technique for visualizing high-dimensional data by reducing its dimensionality for representation in 2D plots. It achieves this by projecting data from a high-dimensional space to a lower-dimensional one, optimizing the placement of points to align the probability distributions of pairwise similarities. This process preserves both the local and global structure of the data.

We used t-SNE to visualize test dataset cell images cropped, segmented, and tracked by our models. The aim was to uncover potential relationships or patterns within the data. Feature vectors were extracted from the processed cells using a custom encoder UNet specifically trained on this dataset. These feature vectors were then used to generate a t-SNE plot.

## Results

### YOLOv8x outperforms YOLOv8l in object detection with an optimal threshold of 0.65

We evaluated the performance of the YOLOv8 models using four performance metrics: mean average precision at an Intersection over Union (IoU) threshold of 0.50 (mAP50), mean average precision across an IoU range of 0.5 to 0.95 (mAP50-95), precision, and recall. The YOLOv8x model consistently demonstrated slightly superior performance across all these metrics compared to the YOLOv8l model when detecting cell bounding boxes in the CELLULAR dataset (see S3 Table in Supplementary Material). A confidence threshold of 0.65 was chosen because it yielded the most accurate results, with the model detecting a cell count closest to the actual number. This threshold filters out low-confidence predictions, keeping only reliable detections.

### Enhanced cell segmentation after fine-tuning and combination with object detection algorithms

Although fine-tuning improved the performance of SAM and Cellpose for cell segmentation, a high number of cells were still incorrectly segmented (see S4 Table in Supplementary Material). To address this, we segmented cropped images delineated by bounding boxes. We compared three segmentation algorithms on these cropped images: SAM, DeepLabV3+, and U-Net++, evaluating them on IoU, F1 score, precision, recall, and accuracy. The binary segmentation results are presented in Table 1, with U-Net++ achieving the best overall performance.

**Table 1:** Comparison of segmentation outcomes between pre-trained and fine-tuned MedSAM, DeepLabV3+, and U-Net++ models applied to cropped cell images using bounding boxes.

		IoU	F1 score	Precision	Recall	Accuracy
**MedSAM**	pre-trained	0.66	0.79	**0.91**	0.71	0.77
	fine-tuned	0.73	0.84	0.86	0.83	0.8
**DeepLabV3+**	from scratch	0.81	**0.9**	0.83	**0.98**	0.85
**U-Net++**	from scratch	**0.82**	**0.9**	0.84	0.97	**0.86**

Overall, segmenting cropped images with cells previously detected significantly enhances segmentation accuracy compared to applying segmentation models directly to whole images containing multiple cells.

### Traditional experimental analysis demonstrate autophagy induction in mRFP-EGFP-Atg8a S2 cells during starvation

**Fig 3:**
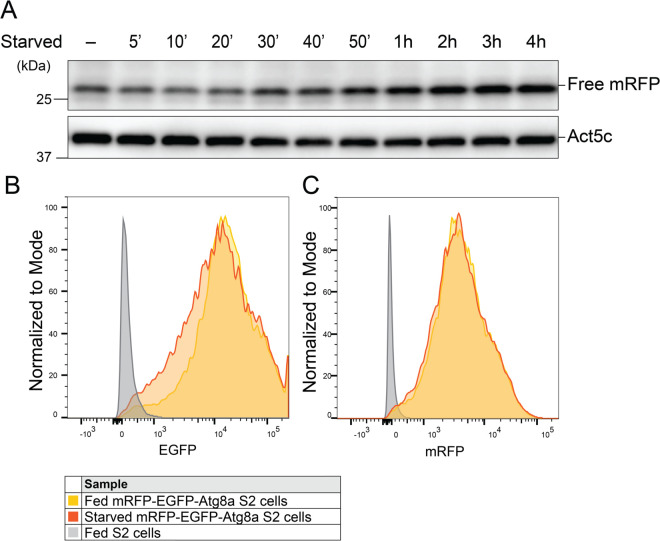
Starvation induces an autophagic response in S2 cell. A: Western blot showing free mRFP levels from mRFP-EGFP-Atg8a S2 cells that were kept fed or starved for the indicated times. *β*-actin/Act5C was used as a loading control. B and C: Fed (yellow) and starved (orange) mRFP-EGFP-Atg8a S2 cells that were subjected to flow cytometry to detect cells with green (B) or red fluorescence (C). S2 cells with no reporter expression were used as negative control.

To validate that mRFP-EGFP-Atg8a S2 cells have an autophagic response upon starvation, we performed Western blot and Flow cytometry analyses. In the Western blot assay, we took advantage of the fact that the tightly folded fluorescent proteins mRFP and EGFP are relatively resistant to lysosomal proteolysis, whereas the full-length mRFP-EGFP-Atg8a fusion protein is efficiently degraded into smaller fragments during autophagy. As autophagic flux increases, free fluorescent proteins accumulate in autolysosomes and can be detected by immunoblotting. Consistent with this, the Western blot analysis showed that free mRFP levels increased with starvation and continued to accumulate over the 4-hour starvation period (Fig 3). In parallel, flow cytometry analysis measures fluorescence intensities of both mRFP and EGFP to assess lysosomal quenching in response to starvation. As EGFP is quenched more rapidly in autolysosomes than mRFP, due to their different pKa values, we can use this to detect the formation of autolysosomes, indicating flux through the autophagy pathway. This analysis demonstrated a reduction in EGFP fluorescence intensity following 1 hour of starvation compared to the fed condition. In contrast, mRFP fluorescence intensity remained largely unchanged between conditions, with overlapping and comparable distribution. Overall, these findings demonstrated that the mRFP-EGFP-Atg8a S2 cells activate autophagy in response to starvation.

### Traditional image analysis fails to classify autophagy at single cell level

**Fig 4:**
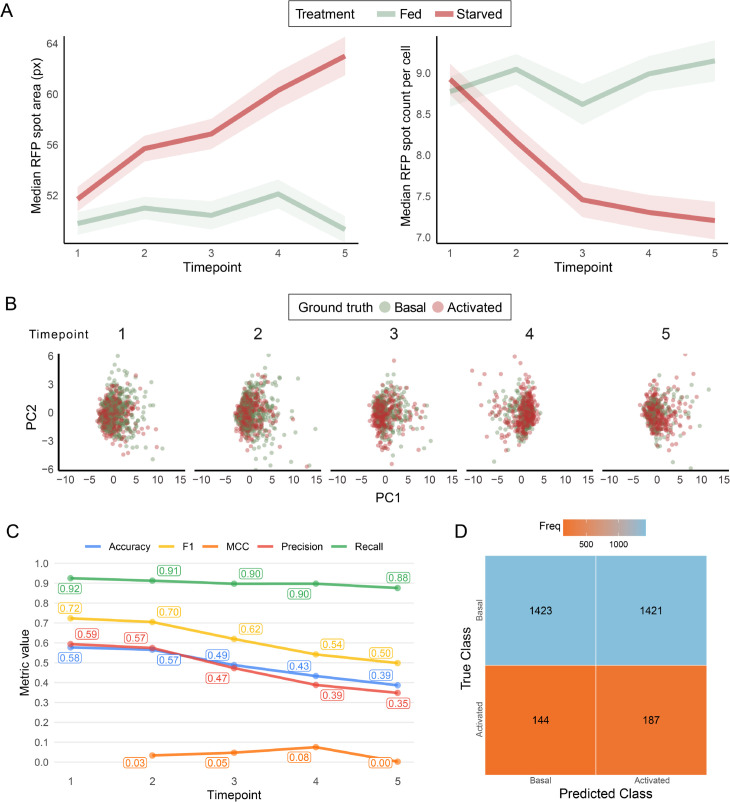
Traditional image analysis of the CELLULAR dataset. A: Spot area and spot count of mRFP-EGFP-Atg8a spots segmented based on the RFP channel in the annotated CELLULAR data set. B: PCA plots of the individual cells based on the ground truth annotations. C: Metrics for classification based on the activated autophagy threshold calculated from the 95th percentile of RFP spot area from fed cells at timepoint 1. D: Confusion matrix for the classification as in C.

Traditional image analysis revealed that RFP spots were generally larger in the cells that had been exposed to starvation, while the total count of RFP spots decreased (Fig 4A). However, separating individual cells based on the ground truth annotations proved difficult as shown by principal component analysis (PCA), indicating limitations in the dimensionality reduction approach of traditional cell features (Fig 4B). To try to classify the cells based on traditional metrics, a threshold for activated autophagy was calculated based on the 95th percentile of RFP spot area from fed cells at timepoint 1. However, the established activated autophagy threshold was ineffective in accurately classifying cells annotated as having activated autophagy, resulting in poor performance (Fig 4C) largely due to overestimation of cells as having basal autophagy (Fig 4D). Furthermore, approximately 11% of the cells did not have RFP spots segmented by the CellProfiler pipeline, resulting in their omission from the analysis. This underscores the need for more unbiased machine learning approaches in the classification of cells undergoing starvation.

### The fine-tuned ViT model achieves the highest accuracy in cell classification based on autophagy status

To overcome the limitations of traditional image analysis methods in classifying autophagy, we investigated deep learning algorithms for classification. The classification of cells based on their autophagy status was evaluated using VGG-16, ResNet50, and ViT models. The cells were categorized into three states: basal autophagy, activated autophagy, and an unidentified category. Fine-tuning these models on our specific dataset of cropped images yielded varying results. The ViT model demonstrated the highest accuracy after fine-tuning, outperforming both VGG-16 and ResNet50 (see Table 2). Fine-tuning significantly enhanced the performance of both ViT and ResNet50, while VGG-16 performed better when trained from scratch. Segmented images consistently led to superior performance across all models (compare Table 2 and S5 Table from Supplementary Material), likely due to reduced background noise and heightened focus on the cells. The fine-tuned ViT model achieved the best accuracy at 86%, followed closely by VGG-16 trained from scratch (see Table 2). Further classification results are discussed in the dedicated explainable AI results section.

**Table 2:** Comparative classification performance using VGG, ResNet, and ViT models, with each model applied in two different training approaches on segmented cells: trained from scratch and fine-tuned.

		F1 score	Precision	Recall	Accuracy	MCC${\;\;}^{a}$
**VGG**	from scratch	0.862	0.863	0.863	0.863	0.790
	fine-tuned	0.843	0.845	0.844	0.844	0.762
**ResNet**	from scratch	0.837	0.837	0.837	0.837	0.751
	fine-tuned	0.860	0.861	0.860	0.860	0.787
**ViT**	from scratch	0.843	0.843	0.844	0.844	0.761
	fine-tuned	**0.863**	**0.863**	**0.864**	**0.864**	**0.792**

a Matthews Correlation Coefficient.

### Visual and interpretive insights confirm model reliability in cell classification

**Fig 5:**
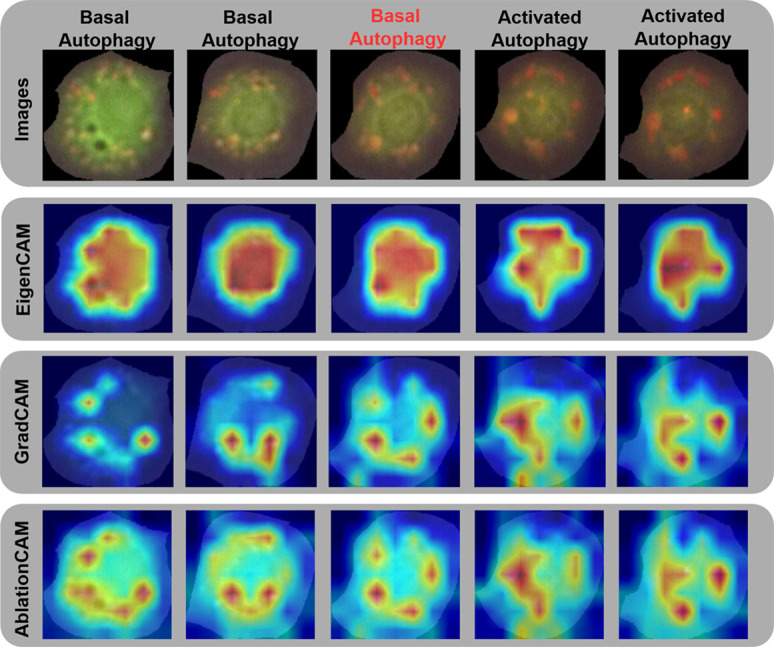
Temporal progression of a starved cell with explainable AI heatmaps. The figure shows a five-time-point sequence from the test dataset, accompanied by GradCAM, EigenCAM, and AblationCAM heatmaps. These highlight key image regions influencing the ViT model’s predictions, with the predicted autophagy status shown above each frame. Notably, an error occurs at time point 3, where basal autophagy is misclassified as activated.

After completing detection, segmentation, classification, and tracking, the best-performing models were evaluated on the test samples. XAI methods were then applied to the classification results, generating corresponding heatmaps, as shown in Fig 5. The first row of images illustrates the progression of a single starved cell over time. At the first three time points, the classification model identified the cell as being in basal autophagy, while at the last two time points, it classified the cell as being in activated autophagy. However, this is incorrect according to the expert classification, as the cell in the third image is already in activated autophagy. We deliberately selected this misclassified example to investigate what the model focused on during its decision-making process.

The subsequent rows of images display the heatmaps generated using different XAI methods, highlighting areas of focus. Warmer colors, such as red, indicate regions of high importance, while cooler colors represent areas considered less relevant. EigenCAM heatmaps differ from those of GradCAM and AblationCAM, as they highlight the entire cell area as important for the model’s decision. In contrast, the other two methods, being class-specific, focus on finer details, such as autophagosomes (observed as yellow dots in the images) in the earlier time points and autolysosomes (observed as red dots in the images) later, aligning closely with human biological classification.

Moreover, we collaborated closely with biology experts to interpret the visualizations and ensure their biological relevance. The experts confirmed that the heatmaps aligned with their visual interpretations of the images. When manually classifying the autophagy status of cells, biologists count the autolysosomes, which become more prominent in later stages of Fig 5. Larger and darker autolysosomes are considered more significant, as they usually appear in the later stages of starvation. In the last three samples, Grad-CAM and Ablation-CAM heatmaps reveal that the model based its decision on only a few autolysosomes, specifically the larger ones, while ignoring the smaller ones at the opposite end of the cell cytoplasm.

### tSNE allows the visual representation of cells gradually transitioning from basal to activated autophagy

**Fig 6:**
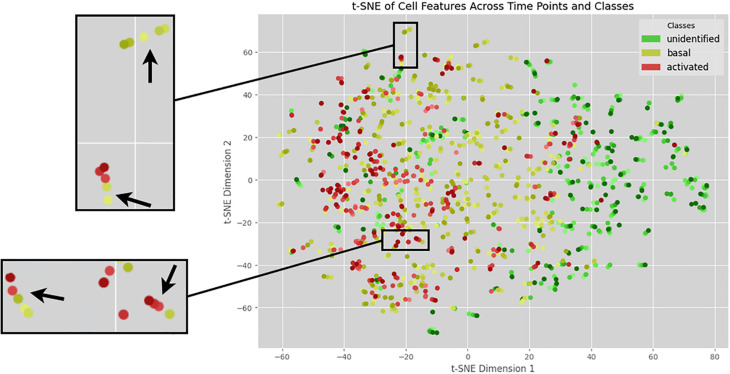
t-SNE plot of processed test data cells. The visualization reflects how cells cluster by class and time. Different colors indicate distinct cell classes, while varying shades within each color represent different time points.

Fig 6 presents the t-SNE plot, where each point represents an individual cell. Green denotes unidentified cells, yellow represents cells with basal autophagy, and red corresponds to cells with activated autophagy. A gradient of colors was applied to indicate the time points, with lighter tones for earlier time points and darker tones for later ones. Clusters of five points can be observed, each representing a single cell tracked across the five time points. To enhance visualization, two enlarged sections highlight these clusters, with arrows indicating each one. Importantly, we observe instances where a cell transitions from basal to activated autophagy, demonstrating the tracking model’s ability to maintain cell identities throughout the sequence. However, the plot also reveals some overlap between cells classified as having basal and activated autophagy, suggesting that these states share similar morphological features. This is expected, as the transition from basal to activated autophagy is a gradual process.

## Discussion

Our study introduces a comprehensive computational pipeline utilizing AI models to automatically detect, segment, classify, track, and interpret cells undergoing autophagic processes, specifically targeting *Drosophila melanogaster* S2 cells. This approach has the potential to significantly accelerate and streamline workflows by replacing the time-consuming and inadequate manual process.

**Fig 7:**
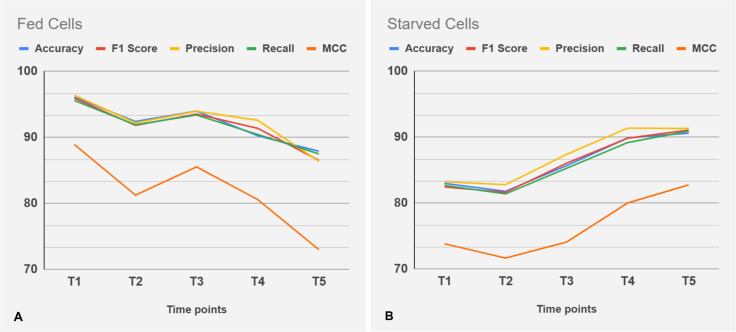
ViT model classification performance over time. Classification results are shown for fed (a) and starved (b) cell sequences across multiple time points in the test dataset. Performance metrics include Accuracy, F1 Score, Precision, Recall, and MCC (Matthews Correlation Coefficient).

Building on this framework, we gained valuable insights into cell classification based on autophagy status. The fine-tuned ViT model achieved an accuracy of 86%, surpassing traditional models such as VGG-16 and ResNet50. Utilizing segmented images significantly contributed to reducing background noise, and enhancing classification outcomes. We further assessed the ViT model’s performance over time on the two test sequences: fed cells (see Fig 7A) and starved cells (see Fig 7B). To ensure consistency across time points, we included only the cells tracked by our algorithm. The model achieved 91% accuracy on fed cells, outperforming its 86% accuracy on starved cells. Most misclassifications in starved cells occurred at time points 1, 2, and 3, which correspond to the transition from basal to activated autophagy. This outcome is expected, as the transition phase is inherently gradual, making it challenging to pinpoint the exact moment a cell shifts from one state to another. Consequently, some level of inconsistency is unavoidable during this phase. In contrast, accuracy improves at time points 4 and 5, when the cells have fully entered the activated autophagy state. Furthermore, the ViT classification metrics outperform the metrics of traditional image analysis algorithms (compare Figs 7A and 7B with Fig 4C).

To enhance model interpretability, we applied explainable AI techniques such as t-SNE and Grad-CAM, offering valuable insights into model performance and decision-making processes. These methods confirmed minimal distortions in cell-class distributions and validated the model’s focus on key cellular structures like autolysosomes. The qualitative validation provided by biology experts highlights the importance of interdisciplinary collaboration. The use of XAI methods is crucial for understanding deep learning models in biomedical research, aligning with observations reported in other studies, such as [32].

**Fig 8:**
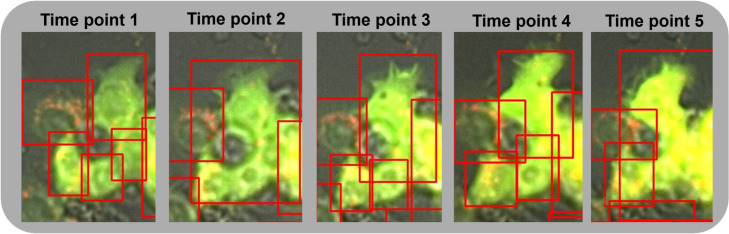
Temporal progression with expert-annotated bounding boxes. The figure illustrates annotation inconsistencies across five time points. While a single cell is identified at time points 2 and 5, multiple cells are annotated at the other time points, indicating variation in expert interpretation.

The object detection annotations in the dataset, provided by human experts, occasionally exhibited inconsistencies. For example, a single cell might be identified as one entity in one frame but split into multiple cells in the next, or a cell detected in one frame might be completely missing in the subsequent frame. These discrepancies stem from differences in interpretation, as various biologists annotated different images, often without following a sequential order. Fig 8 highlights such inconsistencies, where slight changes in the image focal plane make cell borders appear more or less evident to the human eye. However, this particular cell remains unidentified, making it less relevant for autophagy research. From a computational standpoint, though, these irregularities posed challenges for our tracking algorithm. Future research could focus on reducing the focus on unidentified cells.

However, several limitations should be acknowledged. While our custom tracking algorithm appeared to outperform methodologies such as SORT and DeepSORT in handling cell division and morphological changes, these observations were purely qualitative, as no ground truth data was available for quantitative evaluation. This represents a significant limitation. Future research could address this by developing annotations for cell tracking and incorporating deep learning techniques to predict nuanced motion patterns, thereby improving tracking accuracy, even at lower frame rates. Additionally, alternative metrics could be explored, such as track fragmentation, which counts the number of times a single track is split into multiple parts, or an identity switch metric, which measures how often the identities of two cells are incorrectly swapped.

In the broader context, our study builds upon and extends the work of integrating AI with autophagic studies [33–38]. However, relatively few studies have actually used AI methods with microscopy images of autophagy [39]. For instance, Zhang et al. [39] developed DeepPhagy, a deep learning-based for classifying autophagy in Saccharomyces cerevisiae yeast cells. However, the complexity of the tandem-tag marker (mRFP and EGFP) and its variability of expression in *Drosophila melanogaster* S2 cells in our dataset significantly increased the complexity of our images, necessitating the development of a more sophisticated pipeline. Furthermore, Chica et al. [35], used deep learning techniques to classify yeast cell populations based on their microscopy images. Our approach confirms the feasibility of deep learning frameworks for classifying autophagic status and highlights the need for tailored solutions to address the specific challenges of different cell types and imaging techniques.

The primary goal of this work was to develop and validate a pipeline specifically optimized for Drosophila S2 cells and provide a consistent, well-characterized experimental system. The pipeline was tailored to the unique morphology and fluorescence characteristics of S2 cells expressing mRFP-EGFP-Atg8a. Nevertheless, its foundational components including image preprocessing, detection, segmentation, classification, cell tracking and AI explainability were designed with generalizable principles. We collected new data and tested our pipeline with Drosophila S2 cells from a different dataset where cells had been treated with RNAi. We evaluated the pipeline without any additional training or fine-tuning for key genetic perturbations: RNAi-Atg1 (autophagy inhibition), RNAi-mTor (autophagy induction), and RNAi-Luciferase as a control. As shown in Figure S1 from the supplementary material, the results are consistent with the expected biological outcomes: In the control (RNAi Luciferase), the percentage of cells with activated autophagy increased over time of starvation, whereas autophagy levels were reduced in RNAi-Atg1, and increased in RNAi-mTor. These findings support the robustness and biological validity of our model across conditions. For cell types with similar morphologies and tandem reporter systems, only minimal adaptation may be required. Notably, the classification module relies on detecting autophagosomes and autolysosomes, which are conserved cellular structures, suggesting that with limited retraining on annotated datasets from other cell types, the pipeline could be extended to other cultured animal cells expressing comparable markers. However, differences in cell shape, size, or fluorescence intensity may necessitate parameter adjustments and careful validation. Assessing the broader applicability of this pipeline represents an important direction for future research.

## Conclusion

Our work demonstrates significant progress in autophagy-related cellular analysis through the integration of state-of-the-art AI technologies. We implemented and evaluated deep learning models for effective cell detection, segmentation, and classification. The U-Net++ model achieved the best segmentation performance (IoU = 0.82), while the fine-tuned ViT model was the best for cell classification (accuracy of 86%). These results show the potential of transformer architectures over traditional CNNs. Our custom tracking algorithm effectively follows cells across multiple time points without requiring ground truth annotations. To ensure model interpretability, we employed explainable AI techniques, which highlighted biologically relevant features. Expert validation further confirmed the biological significance of the extracted features, reinforcing the potential of AI-driven approaches in autophagy research.

Our findings support the hypothesis that machine learning methods can effectively identify, segment, and track cells as well as classify their autophagic state, improving efficiency and consistency compared to manual annotations. However, further research is needed to refine model accuracy, improve segmentation, and develop robust tracking metrics or ground-truth data to fully realize the potential of these tools in cellular analysis.

## Supporting information

S1 Table Cell counts across time and classes. Number of cells in basal versus activated autophagy across five time points in both nourished and starved environments.
(PDF)

S2 Table Cell morphology comparison. Summary statistics of cell area and circularity under basal and activated autophagy. Reported values include mean, median, and standard deviation, along with results of t-tests comparing the two conditions. Significant differences were observed for both cell area and circularity (*p*<0.001).
(PDF)

S3 Table Object detection results for the YOLOv8 model. (PDF)

S4 Table Segmentation performance of pre-trained vs. fine-tuned models. Performance of pre-trained and fine-tuned Cellpose and SAM models on full images. Fine-tuning improved all metrics, with SAM showing the best overall results.
(PDF)

S5 Table Classification Performance of CNNs and ViT. Comparison of VGG, ResNet, and ViT models trained from scratch versus fine-tuned on non-segmented cells. Performance is reported using F1 score, precision, recall, accuracy, and MCC.
(PDF)

S1 Fig Percentages of cells with activated autophagy over time in RNAi-treated groups. The plot shows the percentage of cells with activated autophagy across time points for RNAi treatments targeting *Atg1*, *mTor*, and Luciferase (control).
(TIFF)

S1 File Original, uncropped and unadjusted annotated images from Fig 3. The antibodies were detected by chemiluminescence, using SuperSignal West DURA Extended Duration Substrate. The molecular weight marker (ladder) was visualized by overlapping an overexposed image, with the corresponding non-overexposed raw image used for analysis.
(PDF)
