# Supplementary material for: Explainable AI to unveil cellular autophagy dynamics
Source: PLoS One. 2025 Sep 11;20(9):e0331045. doi: 10.1371/journal.pone.0331045 (PMC12425229; doi:10.1371/journal.pone.0331045)
Supplement: S1 Table — Number of cells in basal versus activated autophagy across five time points in both nourished and starved environments. (PDF) [file pone.0331045.s001.pdf]

	Nourished Cells					Cells Under Starvation				
**Time Point**	**1**	**2**	**3**	**4**	**5**	**1**	**2**	**3**	**4**	**5**
**Basal Autophagy**	518	470	473	473	470	299	186	92	30	29
**Activated Autophagy**	38	43	40	39	37	202	368	420	430	417
