# Supplementary material for: Explainable AI to unveil cellular autophagy dynamics
Source: PLoS One. 2025 Sep 11;20(9):e0331045. doi: 10.1371/journal.pone.0331045 (PMC12425229; doi:10.1371/journal.pone.0331045)
Supplement: S2 Table — Summary statistics of cell area and circularity under basal and activated autophagy. Reported values include mean, median, and standard deviation, along with results of t-tests comparing the two conditions. Significant differences were observed for both cell area and circularity (p<0.001). (PDF) [file pone.0331045.s002.pdf]

	Cell Area (px^2)		Cell Circularity	
	**Basal Autophagy**	**Activated Autophagy**	**Basal Autophagy**	**Activated Autophagy**
**Mean**	12,834.67	11,209.52	0.63	0.67
**Median**	11,502.25	10,043.00	0.64	0.68
**Std Dev**	7,181.83	6,029.79	0.13	0.12
**T-statistic**	8.49	—	-12.55	—
**p-value**	$p < 0.001$	—	$p < 0.001$	—
