# Supplementary material for: Explainable AI to unveil cellular autophagy dynamics
Source: PLoS One. 2025 Sep 11;20(9):e0331045. doi: 10.1371/journal.pone.0331045 (PMC12425229; doi:10.1371/journal.pone.0331045)
Supplement: S3 Table — (PDF) [file pone.0331045.s003.pdf]

	**mAP50-95**	**mAP50**	**Precision**	**Recall**
**YOLOv8l**	0.52	0.78	0.77	**0.76**
**YOLOv8x**	**0.54**	**0.8**	**0.8**	**0.76**
