# Supplementary material for: Explainable AI to unveil cellular autophagy dynamics
Source: PLoS One. 2025 Sep 11;20(9):e0331045. doi: 10.1371/journal.pone.0331045 (PMC12425229; doi:10.1371/journal.pone.0331045)
Supplement: S4 Table — Performance of pre-trained and fine-tuned Cellpose and SAM models on full images. Fine-tuning improved all metrics, with SAM showing the best overall results. (PDF) [file pone.0331045.s004.pdf]

		**IoU**	**F1 score**	**Precision**	**Recall**	**Accuracy**
**Cellpose**	pre-trained	0.5	0.66	0.7	0.75	0.67
	fine-tuned	0.65	0.78	0.79	0.79	**0.83**
**SAM**	pre-trained	0.36	0.52	**0.89**	0.39	0.52
	fine-tuned	**0.75**	**0.86**	0.82	**0.91**	0.78
