# Supplementary material for: Explainable AI to unveil cellular autophagy dynamics
Source: PLoS One. 2025 Sep 11;20(9):e0331045. doi: 10.1371/journal.pone.0331045 (PMC12425229; doi:10.1371/journal.pone.0331045)
Supplement: S5 Table — Comparison of VGG, ResNet, and ViT models trained from scratch versus fine-tuned on non-segmented cells. Performance is reported using F1 score, precision, recall, accuracy, and MCC. (PDF) [file pone.0331045.s005.pdf]

		F1 score	Precision	Recall	Accuracy	MCC
**VGG**	from scratch	0.854	0.854	0.855	0.855	0.778
	fine-tuned	0.840	0.842	0.842	0.842	0.758
**ResNet**	from scratch	0.839	0.842	0.841	0.841	0.757
	fine-tuned	0.849	0.851	0.85	0.85	0.771
**ViT**	from scratch	0.821	0.823	0.822	0.822	0.728
	fine-tuned	0.848	0.849	0.849	0.849	0.769
