# Supplementary figures and images for: Explainable AI to unveil cellular autophagy dynamics
Source: PLoS One. 2025 Sep 11;20(9):e0331045. doi: 10.1371/journal.pone.0331045 (PMC12425229; doi:10.1371/journal.pone.0331045)

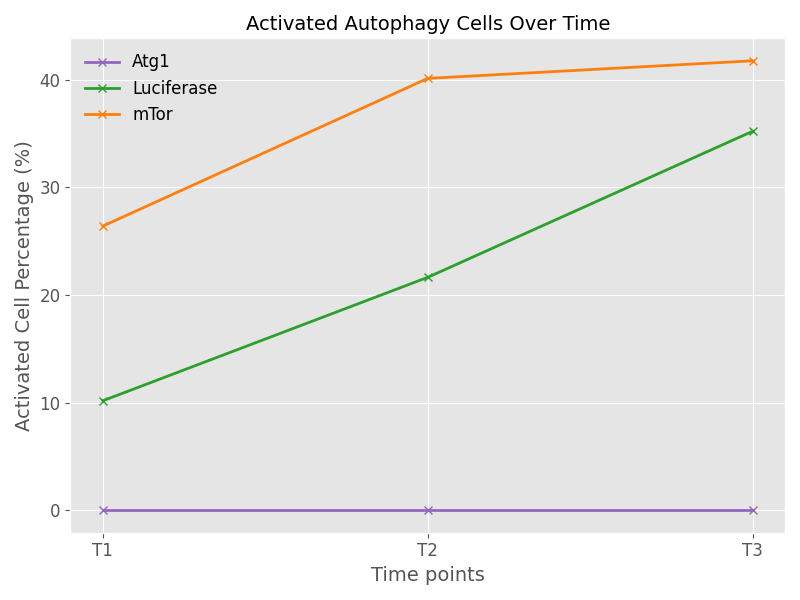

Supplement: S1 Fig — The plot shows the percentage of cells with activated autophagy across time points for RNAi treatments targeting Atg1, mTor, and Luciferase (control). (TIFF) [file pone.0331045.s006.tif]

goat anti-mCherry 1:500 (Acris, AB0040-200)

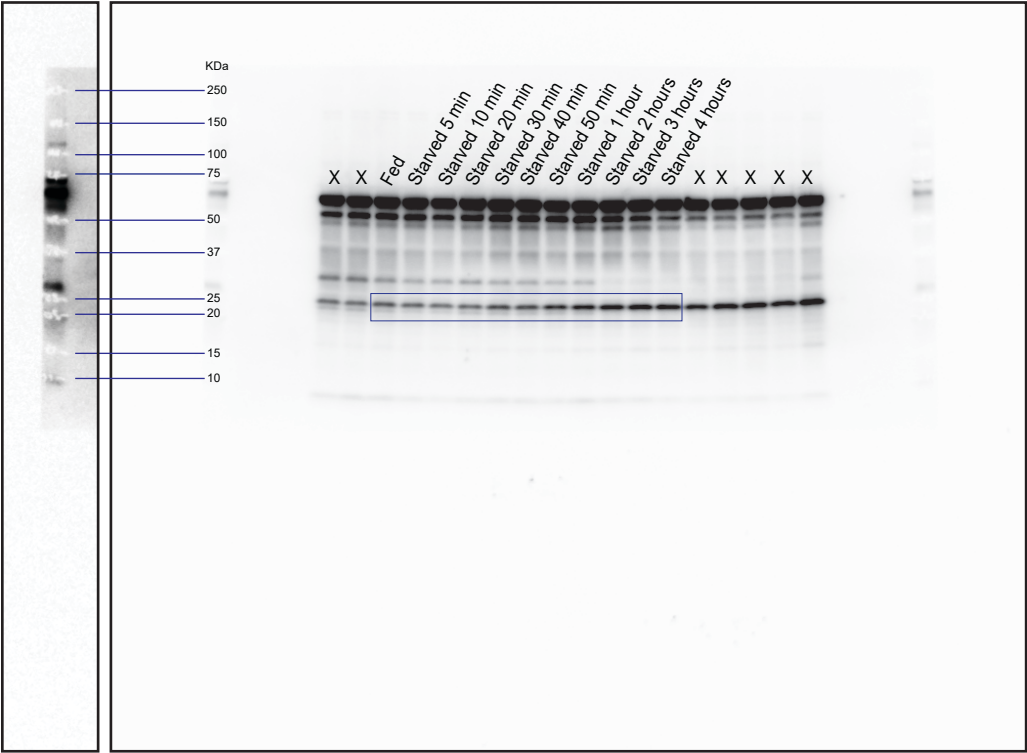

rabbit anti- $\beta$ -actin/Act5C 1:1000 (Abcam, ab8227)

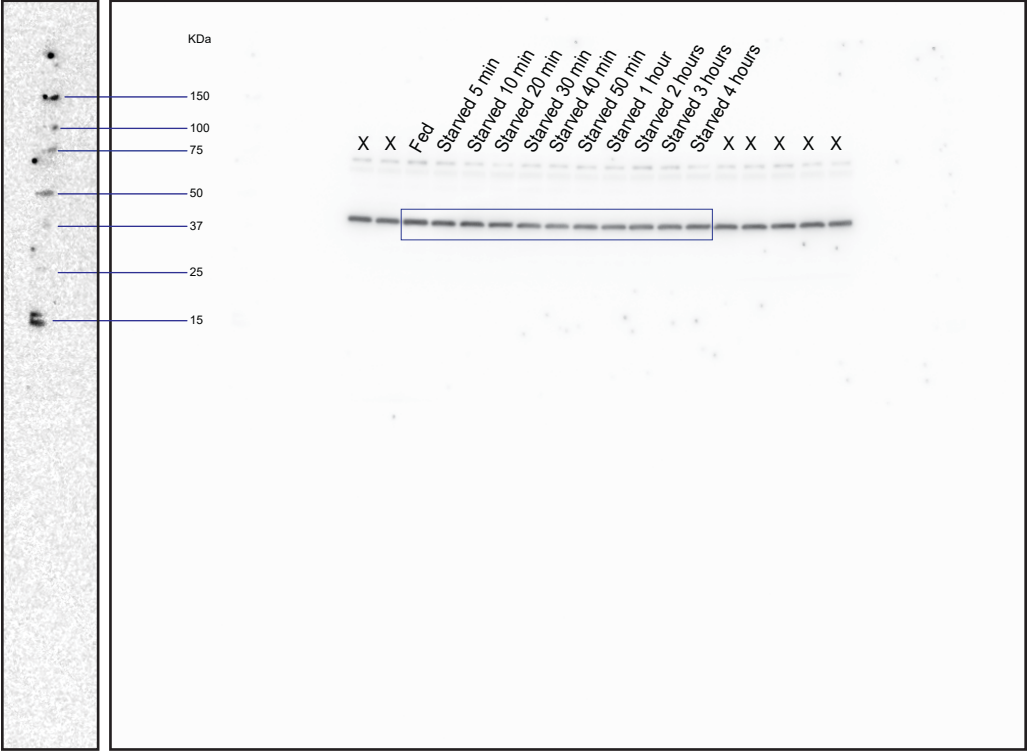

Supplement: S1 File — The antibodies were detected by chemiluminescence, using SuperSignal West DURA Extended Duration Substrate. The molecular weight marker (ladder) was visualized by overlapping an overexposed image, with the corresponding non-overexposed raw image used for analysis. (PDF) [file pone.0331045.s007.pdf]
